# Symptomatic Reinfection in Previously Recovered Coronavirus Disease 2019 (COVID-19) Geriatric Patient

**DOI:** 10.7759/cureus.13961

**Published:** 2021-03-17

**Authors:** Dhigishaba Jadeja, Payel Basak

**Affiliations:** 1 Graduate Medical Education, Gujarat Adani Institute of Medical Sciences, Bhuj, IND; 2 Graduate Medical Education, College of Medicine and Jawaharlal Nehru Memorial (JNM) Hospital, Kalyani, IND

**Keywords:** coronavirus disease, reactivation

## Abstract

Can a patient diagnosed with coronavirus disease 2019 (COVID-19) be infected again? This issue appears to be unsolved. Protective immunity following infection with COVID-19 is still not fully known. In the coming months, an awareness of COVID-19 reinfection will be crucial in directing government and public health policy managements. Here, we present a case of symptomatic reinfection following recovery from COVID-19 in a geriatric patient.

## Introduction

As of February 23, 2021, the novel causative virus, the severe acute respiratory syndrome coronavirus 2 (SARS-CoV-2), has affected 11,030,176 people and caused 156,567 deaths, with a case fatality rate (CFR) of 1.41 percentage (%) in India, which was 1.60% on September 22, 2020 [1]. Gao et al. reported the prevalence of recurrent SARS-CoV-2 17% in previously infected COVID-19 patients [2]. The pandemic is becoming more complicated, and disease prevention, both in terms of morbidity and mortality rates, is becoming more difficult. In addition to the two negative real-time reverse-transcription polymerase chain reaction (RT-PCR) tests for SARS-CoV-2 drawn at least 24 hours apart, the patients infected with coronavirus disease 2019 (COVID-19) may easily discontinue home isolation and are deemed non-infectious after complete symptomatic recovery. Current literature is buzzing with the symptomatic reinfection in patients who previously recovered from COVID-19 [3]. Risk factors for the reactivation of SARS-CoV-2 might be associated with the type of immunosuppressive treatment, host factors such as the elderly, ethnicity, underlying diseases such as diabetes, cancer, obesity, cardiac disease, virological factors, weak immune response due to non-invasive infection, inadequate monitoring of SARS-CoV-2 infection, an inflammatory reaction that has resurfaced as a consequence of an inappropriate immune response, and different viral strains [4,5]. While infection with the SARS-CoV-2 results in a visible immune response, it is unclear if previously infected individuals are susceptible to reinfection with SARS-CoV-2. Infection with SARS-CoV-2 causes the development of neutralizing antibodies in patients [6]. However, the extent to which this immune response suggests defensive immunity against subsequent SARS-CoV-2 infection is yet to be determined. Here, we present a case of symptomatic reinfection in a recovered COVID-19 geriatric patient.

## Case presentation

A 79-year-old male presented in the emergency room with cough, breathlessness, and fever (38.5°C). The patient's past medical history was remarkable for chronic ischemic heart disease and smoking.

The patient’s recent clinical history had begun on July 9, 2020 with the appearance of a fever (27.9 °C), dry cough, breathlessness. On the 14th day of illness, with the emergence of dyspnea, there was a deterioration of clinical symptoms and so the patient was hospitalized in the COVID care center. There, he tested positive for SARS-CoV-2 by RT-PCR from a nasopharyngeal swab, and his chest computed tomography (CT) scan revealed bilateral infiltrates. During the hospital course, he was treated with favipiravir 1600 milligram (mg) twice daily followed by 600 mg twice daily for seven days and hydroxychloroquine 400 mg twice daily on first day followed by 200 mg twice daily for another four days, together with supportive oxygen with the nasal cannula at flow 1.5 liters/minute (L/m).

By the 18th day of illness, respiratory symptoms had resolved. Laboratory biomarkers were negative including C-reactive protein (CRP), positive immunoglobulin G (IgG) antibody, and negative immunoglobulin M (IgM) antibody for COVID-19, and nasopharyngeal swabs for SARS-CoV-2 were taken serially two times, resulting in negative RT-PCR. The patient was deemed at home to be healed and discharged.

**Figure 1:**
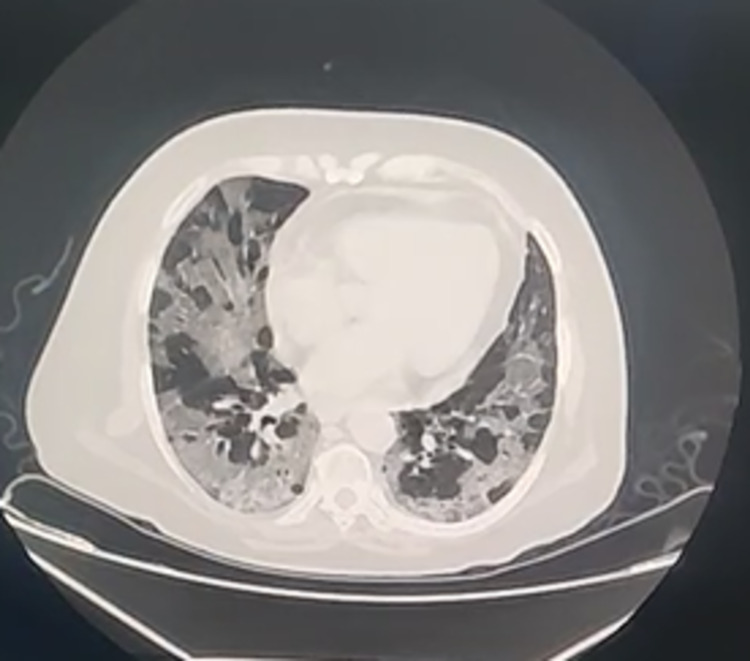
Computed tomography (CT) findings in recovered COVID-19 patient

On August 17, 2020, he again developed symptoms in the form of intermittent 38°C fever, generalized body ache, throat pain, and his rapid antigen test (RAT) returned positive. On August 19, 2020, the patient needed rehospitalization where his COVID-19 RT-PCR returned positive with a cycle threshold (CT) score of 17 indicative of high viral load, confirming a case of COVID-19 reinfection (Figure 1).

His inflammatory profile revealed CRP of 52.8 mg/L and IL-6 of 20.4 picograms per milliliter (pg/mL). After discussion with the COVID care team, the patient was treated symptomatically. After eight days, the patient became afebrile and no respiratory symptoms observed since then.

## Discussion

SARS-CoV-2 is characterized by hyper-cytokinemia, which typically occurs in the second week of COVID-19 and is associated with immunodeficiency as well as hyper inflammation, the latter manifesting as a cytokine storm [7]. COVID-19 was earlier thought to be a respiratory disease, but it has affected multiple organs [8-11]. A study done by Desai et al. reported that COVID-19 has caused not only physical distress but also had a negative impact on social, economic, and psychological well-being [12]. At present, literature is buzzing with the symptomatic reinfection of SARS-CoV-2 in recovered COVID-19 patients. Previous exposure to SARS-CoV-2 does not necessarily result in total immunity being guaranteed. A study done by Tillett et al. on genomic analysis of SARS-CoV-2 revealed genetically significant variations between each variant associated with each infection case. Furthermore, they also reported that the second infection was clinically more severe than the first and severity might be associated with the viral load, the virulence of the viral strain [13]. A study done by Song et al. has reported that the post-negative positive RT-PCR findings may be due to the identification of ribonucleic acid (RNA) particles rather than reinfection in individuals that have recovered from COVID-19 [14]. Studies have shown that the humoral response can be poor in patients who are asymptomatic at the time of the post-negative positive RT-PCR test and may improve progressively. In all COVID-19 patients, however, IgM and IgG become detectable between the third and fourth weeks of the onset of their clinical disease [6,15]. In our case, IgG antibody was found to be positive while IgM antibody was negative for COVID-19. The confirmation of re-infection has several important implications. First, herd immunity is unlikely to eradicate SARS-CoV-2, although it might make future infections milder than the first infection. Second, the probability of COVID-19 reinfection indicates that, as with other human coronaviruses, COVID-19 could become widespread in some pockets of seasonal outbreaks. Third, vaccinations may not be sufficient to offer a lifetime defense against COVID-19, and even though we create a vaccine, sustainable immuno-protection may require booster dosing. Fourth, vaccine studies may have to be performed in such patients who have recovered from COVID-19 [16]. A vaccination that stimulates cross-protective immunity will be an effective tool for preventing or reducing the severity of disease caused by potential pandemic coronavirus strains. As a result, even if reinfection happens when antibody levels are low, pre-existing T-cell immunity can deter clinically serious disease [17,18]. SARS-CoV-2 epitopes have been identified in previously circulating human coronaviruses, indicating that they may play a role in the immune defense of COVID-19 unexposed and recovering patients [19]. Furthermore, telemedicine may help to reduce the severity of the infection and prompt identification of recurrence cases in patients with comorbidities [20]. In India and around the world, the lack of systematic genomic sequencing of positive cases restricts the advancements in public health monitoring needed to find these cases. Limitations in SARS-CoV-2 screening and testing worsen the ineffective surveillance attempts being made not only to detect COVID-19 but also to achieve actionable genetic monitoring of this agent.

## Conclusions

This case of reinfection shows that population transmission herd immunity could be an elusive tactic and the production of vaccines needs to be reoriented towards potential single infection weaning immunity. People must take precaution to prevent SARS-CoV-2 infection irrespective of whether they diagnosed previously or not from the public health perspective. Future studies need to focus on immune responses in vitro after reinfection. 
